# Alterations of Functional and Structural Networks in Schizophrenia Patients with Auditory Verbal Hallucinations

**DOI:** 10.3389/fnhum.2016.00114

**Published:** 2016-03-15

**Authors:** Jiajia Zhu, Chunli Wang, Feng Liu, Wen Qin, Jie Li, Chuanjun Zhuo

**Affiliations:** ^1^Department of Radiology and Tianjin Key Laboratory of Functional Imaging, Tianjin Medical University General HospitalTianjin, China; ^2^Department of Psychiatry Functional Neuroimaging Laboratory, Tianjin Anding Hospital, Tianjin Mental Health CenterTianjin, China; ^3^Tianjin Anning HospitalTianjin, China

**Keywords:** schizophrenia, auditory verbal hallucinations, functional magnetic resonance imaging, diffusion tensor imaging, small-world network

## Abstract

**Background:** There have been many attempts at explaining the underlying neuropathological mechanisms of auditory verbal hallucinations (AVH) in schizophrenia on the basis of regional brain changes, with the most consistent findings being that AVH are associated with functional and structural impairments in auditory and speech-related regions. However, the human brain is a complex network and the global topological alterations specific to AVH in schizophrenia remain unclear.

**Methods:** Thirty-five schizophrenia patients with AVH, 41 patients without AVH, and 50 healthy controls underwent resting-state functional magnetic resonance imaging (fMRI) and diffusion tensor imaging (DTI). The whole-brain functional and structural networks were constructed and analyzed using graph theoretical approaches. Inter-group differences in global network metrics (including small-world properties and network efficiency) were investigated.

**Results:** We found that three groups had a typical small-world topology in both functional and structural networks. More importantly, schizophrenia patients with and without AVH exhibited common disruptions of functional networks, characterized by decreased clustering coefficient, global efficiency and local efficiency, and increased characteristic path length; structural networks of only schizophrenia patients with AVH showed increased characteristic path length compared with those of healthy controls.

**Conclusion:** Our findings suggest that less “small-worldization” and lower network efficiency of functional networks may be an independent trait characteristic of schizophrenia, and regularization of structural networks may be the underlying pathological process engaged in schizophrenic AVH symptom expression.

## Introduction

Hallucinations, which are defined as any perceptual experience in the absence of corresponding stimuli in the external world (David, 2004), are one of the most debilitating symptoms of schizophrenia. Although they may involve any of the senses, auditory verbal hallucinations (AVH) or “hearing voices” are the most prevalent type of hallucinations in schizophrenia (Andreasen and Flaum, 1991), and affect about 60–80% of schizophrenic patients. In about 25% of these patients, AVH are chronic and resistant to medication (Shergill et al., 1998), causing an impaired quality of life (Gaite et al., 2002).

In the past two decades, the advent of neuroimaging techniques has allowed researchers to explore the neurobiological basis of schizophrenic AVH *in vivo* (Allen et al., 2008, 2012). Research into brain structural and functional alterations [including gray matter volume (Allen et al., 2008, 2012; Palaniyappan et al., 2012; Modinos et al., 2013), cortical thickness (van Swam et al., 2012), white matter integrity (Geoffroy et al., 2014; Wigand et al., 2015), state activation (Jardri et al., 2011; Kompus et al., 2011; Allen et al., 2012; Kuhn and Gallinat, 2012), and resting-state functional connectivity (Hoffman and Hampson, 2011)] related to AVH has been accumulating in recent years. Some key brain regions involving auditory processing, speech generation and speech perception have been identified. These AVH-related brain regions include the superior temporal gyrus, Wernicke’s area, Broca’s area, and arcuate fasciculus bundle. However, previous studies only focus on regional changes of the brain, and it is largely unknown whether there are global topological alterations specific to AVH in schizophrenia.

Recently, an increasing number of brain network studies based on graph theory have emerged using modern brain mapping techniques, such as functional magnetic resonance imaging (fMRI) and diffusion tensor imaging (DTI; Bullmore and Sporns, 2009). The application of graph theoretical approaches has provided a powerful tool to characterize topological properties of complex brain networks on a whole brain scale by defining a graph as a set of nodes (brain regions) and edges (functional or structural connections; Bullmore and Sporns, 2009; He and Evans, 2010; Bullmore and Bassett, 2011). Through these approaches, both structural and functional networks of human brain have been found to have a optimum small-world topology (Watts and Strogatz, 1998), characterized by a high local specialization and a high global integration between brain regions (Salvador et al., 2005; Achard et al., 2006; Bassett and Bullmore, 2006; Bassett et al., 2006; Hagmann et al., 2007; He et al., 2007). Global measures of brain networks are often represented in multiple ways, such as small-world properties and network efficiency, which variously quantify the capability of integration and segregation of information processing, test the global efficiency of parallel information transfer, and characterize the fault tolerance of the network (Rubinov and Sporns, 2010). There is an expanding body of evidence that patients with schizophrenia exhibited disrupted functional and structural brain networks, predominantly characterized by altered global topological properties (Fornito et al., 2012; Micheloyannis, 2012; van den Heuvel and Fornito, 2014).

Because AVH are one of the most characteristic and frequent symptoms with definite brain damage in schizophrenia patients, we hypothesized that there would be abnormality specific to AVH in global topological properties of brain networks. To test our hypothesis, we collected resting-state fMRI and DTI data from 35 schizophrenia patients with AVH, 41 patients without AVH, and 50 healthy controls. Functional and structural networks were constructed and analyzed using graph theoretical approaches. Inter-group differences in global metrics of both networks were investigated.

## Materials and Methods

### Subjects

A total of 126 individuals were enrolled in the present study, including 76 patients with schizophrenia and 50 healthy controls. The diagnoses of schizophrenia were determined by the consensus of two experienced clinical psychiatrists using the Structured Clinical Interview for the DSM-IV Axis I Disorder, Patient Edition (SCID-P). All healthy controls were screened using the non-patient edition of the SCID (SCID-NP) to confirm an absence of psychiatric illnesses. The inclusion criteria for all participants were age (20–60 years) and right-handedness. The exclusion criteria for all participants were a history of head trauma with consciousness disturbances lasting more than 5 min, a history of drug or alcohol abuse, pregnancy, and any physical illness such as cardiovascular disease or neurological disorders, as diagnosed by an interview and medical records review. Additional exclusion criteria for healthy controls included a history of psychiatric disease and first-degree relatives who had a history of psychotic episodes. According to the experience of AVH, schizophrenia patients were subdivided into two groups: AVH group (*n* = 35) included patients who experienced AVH at least once daily, and nAVH group (*n* = 41) included patients who had never experienced AVH or had not experienced AVH within 12 months prior to MRI scanning. Clinical symptoms of psychosis were quantified using the Positive and Negative Syndrome Scale (PANSS; Kay et al., 1987). The Auditory Hallucination Rating Scale (AHRS; Hoffman et al., 2003) was used to assess AVH on seven characteristics: frequency, reality, loudness, number of voices, length, attention dedicated to the hallucinations, and hallucination-induced arousal. The study was conducted in accordance with the Declaration of Helsinki and was approved by the Medical Research Ethics Committee of Tianjin Medical University General Hospital. After a complete description of the study, written informed consent was obtained from each subject.

### MRI Data Acquisition

MRI data were acquired using a 3.0-Tesla MR system (Discovery MR750, General Electric, Milwaukee, WI, USA). Tight but comfortable foam padding was used to minimize head motion, and earplugs were used to reduce scanner noise. 3D T1-weighted images were acquired using a brain volume (BRAVO) sequence with the following parameters: repetition time (TR) = 8.2 ms; echo time (TE) = 3.2 ms; inversion time (TI) = 450 ms; flip angle (FA) = 12°; field of view (FOV) = 256 mm × 256 mm; matrix = 256 × 256; slice thickness = 1 mm, no gap; 188 sagittal slices; and acquisition time = 250 s. Resting-state functional blood-oxygen-level-dependent (BOLD) images were acquired using a gradient-echo single-short echo planar imaging (GRE-SS-EPI) sequence with the following parameters: TR/TE = 2000/45 ms; FOV = 220 mm × 220 mm; matrix = 64 × 64; FA = 90°; slice thickness = 4 mm; gap = 0.5 mm; 32 interleaved transverse slices; 180 volumes; and acquisition time = 370 s. DTI data were acquired by a spin-echo single-shot echo planar imaging (SE-SS-EPI) sequence with the following parameters: TR/TE = 5800/77 ms; FOV = 256 mm × 256 mm; matrix = 128 × 128; in-plane resolution = 2 mm × 2 mm; slice thickness = 3mm without gap; 48 axial slices; 25 non-collinear diffusion gradients (b = 1000 s/mm^2^) and 10 non-diffusion-weighted images (b = 0 s/mm^2^); and acquisition time = 354 s. All subjects were instructed to keep their eyes closed, relax, move as little as possible, think of nothing in particular, and not fall asleep during the scans. All MR images were visually inspected to ensure that only images without visible artifacts were included in subsequent analyses.

### fMRI Data Preprocessing

Resting-state BOLD data were preprocessed using Statistical Parametric Mapping 8 (SPM8^1^). The first 10 volumes for each participant were discarded to allow the signal to reach equilibrium and the participants to adapt to the scanning noise. The remaining volumes were corrected for the acquisition time delay between slices. Then, realignment was performed to correct the motion between time points. All participants’ BOLD data were within the defined motion thresholds (i.e., translational or rotational motion parameters less than 2 mm or 2°). We also calculated frame-wise displacement (FD), which indexes the volume-to-volume changes in head position. There were no significant differences in mean FD (one-way ANOVA, *F* = 0.421, *P* = 0.657) among the AVH (0.106 ± 0.046), nAVH (0.112 ± 0.057), and control (0.102 ± 0.052) groups. Several nuisance covariates (six motion parameters, their first time derivations, the global brain signal, the white matter signal, and the cerebrospinal fluid signal) were regressed out from the data. Recent studies have reported that the signal spike caused by head motion significantly contaminated the final resting-state fMRI results even after regressing out the linear motion parameters (Power et al., 2012). Therefore, we further regressed out spike volumes when the FD of the specific volume exceeded 0.5. The datasets were then band-pass filtered in a frequency range of 0.01–0.08 Hz. In the normalization step, individual structural images were firstly co-registered with the mean functional image; then the transformed structural images were segmented and normalized to the Montreal Neurological Institute (MNI) space using a high-level non-linear warping algorithm, that is, the diffeomorphic anatomical registration through the exponentiated Lie algebra (DARTEL) technique (Ashburner, 2007). Finally, each filtered functional volume was spatially normalized to MNI space using the deformation parameters estimated during the above two steps and resampled into a 3-mm cubic voxel.

### Functional Network Construction

**FIGURE 1:**
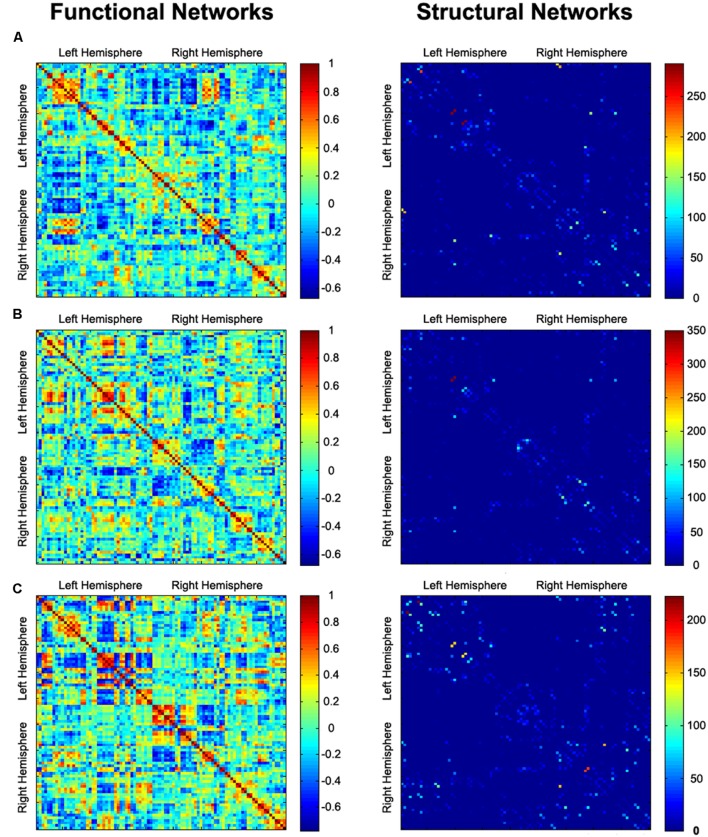
**Functional connectivity (correlation coefficient) and structural connectivity (fiber number) matrices for the construction of functional and structural weighted networks for AVH **(A)**, nAVH **(B)**, and HC **(C)**.** Both axes represent the 90 AAL brain regions used in the analysis. Color of each entry represents the level of connectivity. AVH, schizophrenia patients with auditory verbal hallucinations; nAVH, schizophrenia patients without auditory verbal hallucinations; HC, healthy controls.

GRETNA software^2^ was used to construct the whole-brain networks (Wang et al., 2015b). Nodes and edges are two basic elements of a brain network. To define the nodes of brain networks, we used the automated anatomical labeling (AAL) template (Tzourio-Mazoyer et al., 2002) to parcellate the whole brain into 90 (45 for each hemisphere) cortical and subcortical regions of interest (ROIs). For each subject, the representative mean time series of each ROI was obtained by averaging the BOLD time series over all voxels within that region. To define the edges of functional brain networks, we computed the Pearson correlation coefficients and their significance levels (i.e., *P*-values) between the regional mean time series of all possible pairs of nodes, resulting in a 90 × 90 correlation matrix for each subject (**Figure 1**). Because of the ambiguous interpretation of negative correlations (Fox et al., 2009; Murphy et al., 2009), we restricted our analysis to positive correlations. To further de-noise spurious interregional correlations, only those correlations whose corresponding significance levels survived a statistical threshold of *P* < 0.05 (Bonferroni corrected) were retained. This significance level-defined correlation threshold method has been used in previous brain network studies (Bassett et al., 2011; Wang et al., 2013, 2015a). Finally, the functional weighted networks were constructed with considering functional connectivity as the weight of each edge. To test the effects of the choices of correlation thresholds on our network analyses, we also constructed the functional networks using other two different correlation thresholds, that is, a looser one whose corresponding significance level survived a statistical threshold of *P* < 0.001 (uncorrected) and a stricter one whose corresponding significance level survived a statistical threshold of *P* < 0.01 (Bonferroni corrected).

### DTI Data Preprocessing

The software packages FMRIB Software Library (FSL^3^) and Diffusion Toolkit (DTK^4^) were used for the DTI preprocessing steps. The diffusion-weighted images were first registered to a reference volume (i.e., the first b0 image) by using affine transformations to minimize distortions caused by the eddy currents and head motions. Then, the three-dimensional maps of the diffusion tensor and the fractional anisotropy (FA) were calculated by using DTK. The whole-brain deterministic fiber tracking was performed via Fiber Assignment by Continuous Tracking (FACT) algorithm (Mori et al., 1999) with the FA threshold of 0.2 and the angle threshold of 50°.

### Structural Network Construction

Because structural networks were constructed in the diffusion image native space, the AAL template in MNI space was needed to be transformed to individual native space. Briefly, individual structural images were firstly co-registered to their first b0 images. Then the transformed structural images were segmented and normalized to the MNI space using a non-linear warping algorithm, i.e., the DARTEL technique. The derived deformation parameters were inverted and used to transform the AAL template from MNI space to diffusion image native space. To define the edges of structural brain networks, we calculated the number of fibers (with end-points in both nodes during the fiber tracking) between any pairs of nodes, resulting in a 90 × 90 fiber number (FN) matrix for each subject (**Figure 1**). Considering the limited resolution of DTI and the capacity of the FACT algorithm, there is a risk that some pseudoconnections will be included. To reduce influence of possible pseudoconnections, a threshold of three fibers was applied to all FN matrices, that is, two nodes were considered connected with an edge if at least three fibers existed (Li et al., 2009; Shu et al., 2009; Lo et al., 2010). Finally, the structural weighted networks were constructed with considering FN as the weight of each edge (Li et al., 2009; Bai et al., 2012; Baggio et al., 2015; Zhao et al., 2015).

### Network Analysis

For both functional and structural weighted brain networks, global network metrics were calculated. Small-world properties of the brain networks are the most frequently used metrics, including clustering coefficient *C*p, characteristic path length *L*p, normalized clustering coefficient γ, normalized characteristic path length λ, and small-worldness σ (Watts and Strogatz, 1998). *C*_p_ is a measure of the extent of the local density or cliquishness of the network, which characterizes network segregation. *L*_p_ is a measure of the extent of average connectivity or overall routing efficiency of the network, which characterizes network integration. They were scaled against the mean clustering coefficient and characteristic path length obtained from 1000 matched random networks which had the same number of nodes, edges, and degree distribution as the real networks (Maslov and Sneppen, 2002), resulting in normalized clustering coefficient γ and normalized characteristic path length λ. These two measurements can also be summarized into a simple quantitative metric, small-worldness, σ = γ/λ.

The brain networks have been found to have efficient small-world properties in support of efficient transfer of parallel information at a relatively low cost (Achard and Bullmore, 2007). Thus, network efficiency is a biological relevant metric to describe brain networks from the perspective of information transfer (Latora and Marchiori, 2001; Achard and Bullmore, 2007). We calculated the network efficiency at global and local levels. The global efficiency *E*g represents the capability of parallel information transfer over the network. The local efficiency *E*loc reflects the fault tolerance of the network, which indicates how well the information is communicated within the neighbors of a given node when this node is eliminated. They were also scaled against the mean *E*g and *E*loc obtained from 1000 matched random networks, resulting in normalized *E*g and normalized *E*loc.

### Statistical Analysis

To determine the inter-group differences in the global topological properties of the functional and structural networks, a one-way analysis of variance (ANOVA) were separately performed on each network metric (small-world properties and network efficiency) using non-parametric permutation tests. The non-parametric permutation tests were conducted using MATLAB language and the number of permutations was set to 10000. To examine the relationship between global topological properties and severity of AVH, a Spearmen’s correlation analysis between each network metric and AHRS total score was performed in the AVH group. Because these analyses were exploratory in nature, we used a statistical significance level of *P* < 0.05. To validate our main findings, the same ANOVA was applied to network metrics of the functional networks constructed at the other two correlation thresholds (*P* < 0.001, uncorrected and *P* < 0.01, Bonferroni corrected), respectively.

## Results

### Demographic and Clinical Characteristics

Demographic and clinical data for the subjects are presented in **Table 1**. The three groups were well-matched in gender (chi-square test, χ^2^ = 0.308, *P* = 0.857) and age (one-way ANOVA, *F* = 0.100, *P* = 0.905). There were no significant differences in antipsychotic dosages (two sample *t*-test, *t* = 1.163, *P* = 0.248), durations of illness (two sample *t*-test, *t* = -0.916, *P* = 0.363), PANSS negative score (two sample *t*-test, *t* = -0.594, *P* = 0.555), PANSS general score (two sample *t*-test, *t* = 0.275, *P* = 0.784) and PANSS total score (two sample *t*-test, *t* = 0.893, *P* = 0.375) between AVH and nAVH groups.

**Table 1:** Demographic and clinical characteristics of the participants.

Characteristics	AVH (*n* = 35)	nAVH (*n* = 41)	HC (*n* = 50)	*P*-value
Age (years)	31.5 ± 7.7	32.3 ± 5.7	32.0 ± 8.2	0.905^a^
Sex (female/male)	17/24	14/21	18/32	0.857^b^
Antipsychotic dosage (mg/d; chlorpromazine equivalents)	518.1 ± 395.6	429.9 ± 259.5	NA	0.248^c^
Duration of illness (months)	101.4 ± 94.3	118.8 ± 71.7	NA	0.363^c^
PANSS				
Total	73.2 ± 23.6	68.4 ± 23.0	NA	0.375^c^
Positive score	20.1 ± 7.7	14.8 ± 7.6	NA	0.004^c^
Negative score	18.8 ± 8.2	20.2 ± 9.2	NA	0.555^c^
General score	34.3 ± 11.5	33.6 ± 10.5	NA	0.784^c^
AHRS total score	23.9 ± 8.4	NA	NA	


*The data are shown as the mean values ± standard deviations. NA, not applicable; PANSS, The Positive and Negative Syndrome Scale; AHRS, The Auditory Hallucination Rating Scale; AVH, schizophrenia patients with auditory verbal hallucinations; nAVH, schizophrenia patients without auditory verbal hallucinations; HC, healthy controls. ^a^One-way ANOVA was used to test the difference in age across the three groups. ^b^Chi-square test was used to test the difference in gender across the three groups. ^c^Two-sample t-test was used to compare the differences in antipsychotic dosage, duration of illness and PANSS scores between two patient groups.*

### Intergroup Differences in Global Metrics of Functional Networks

**FIGURE 2:**
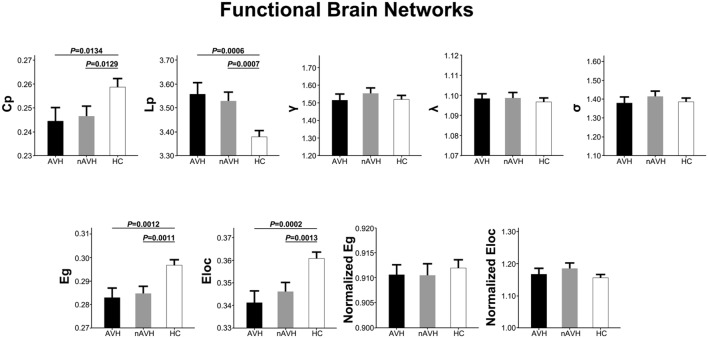
**The differences in global metrics of the functional weighted networks across the AVH, nAVH, and HC groups.** Error bars represent standard errors. *C*p, clustering coefficient; *L*p, characteristic path length; γ, normalized clustering coefficient; λ, normalized characteristic path length; σ, small-worldness; *E*g, global efficiency; *E*loc, local efficiency; AVH, schizophrenia patients with auditory verbal hallucinations; nAVH, schizophrenia patients without auditory verbal hallucinations; HC, healthy controls.

At the significance level-defined correlation threshold (i.e., *P* < 0.05, Bonferroni corrected), functional brain networks of the AVH, nAVH, and control groups exhibited higher clustering coefficients (γ_AV H_ = 1.51 ± 0.21, γ_nAV H_ = 1.55 ± 0.20, γ_control_ = 1.52 ± 0.16) but almost identical characteristic path lengths (λ_AV H_ = 1.10 ± 0.01, λ_nAV H_ = 1.10 ± 0.02, λ_control_ = 1.10 ± 0.01) relative to comparable random networks, which suggests that the three groups had typical small-world properties in functional networks. Despite common small-world architecture of the functional networks, statistical analyses revealed significant differences in global metrics across the AVH, nAVH, and control groups (**Figure 2**). Compared with the control group, functional networks of both AVH and nAVH groups showed significantly decreased *C*p, *E*g, and *E*lo*c*, and increased *L*p. However, no significant differences were observed in *C*p, *L*p, *E*g, and *E*loc between AVH and nAVH groups. In addition, we found that γ, λ, σ, normalized *E*g and normalized *E*loc had no significant differences across the three groups. Moreover, we found that our main results were reproducible after considering the effects of different correlation thresholds (**Supplementary Figures S1** and **S2**). Inter-group differences in basic network properties of functional networks including density, connectivity strength and size of largest component are shown in Supplementary Material (*SI Text*) and **Supplementary Figure S3**.

### Intergroup Differences in Global Metrics of Structural Networks

**FIGURE 3:**
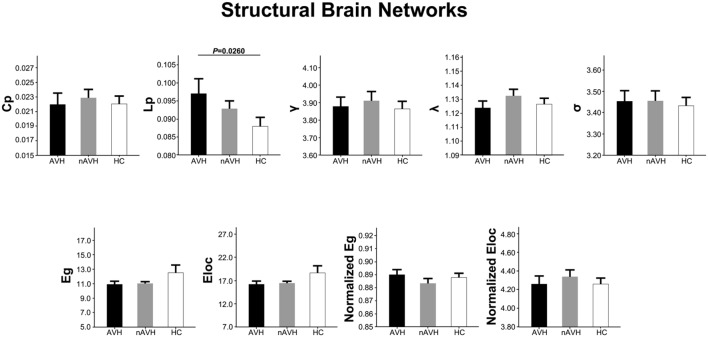
**The differences in global metrics of the structural weighted networks across the AVH, nAVH, and HC groups.** Error bars represent standard errors. *C*p, clustering coefficient; *L*p, characteristic path length; γ, normalized clustering coefficient; λ, normalized characteristic path length; σ, small-worldness; *E*g, global efficiency; *E*loc, local efficiency; AVH, schizophrenia patients with auditory verbal hallucinations; nAVH, schizophrenia patients without auditory verbal hallucinations; HC, healthy controls.

At the threshold of three fibers, structural brain networks of the AVH, nAVH, and control groups had higher clustering coefficients (γ_AV H_ = 3.88 ± 0.32, γ_nAV H_ = 3.91 ± 0.34, γ_control_ = 3.86 ± 0.30) but almost identical characteristic path lengths (λ_AV H_ = 1.12 ± 0.03, λ_nAV H_ = 1.13 ± 0.03, λ_control_ = 1.13 ± 0.03) relative to matched random networks, which indicates that the three groups exhibited a typical small-world topology in structural networks. Differences in global metrics of structural networks across the AVH, nAVH, and control groups are shown in **Figure 3**. Compared with the control group, structural networks of AVH group exhibited significantly increased *L*p. However, there was no significant difference in *L*p between nAVH and control groups. Additionally, we found that *C*p, γ, λ, σ, *E*g, *E*loc, normalized *E*g and normalized *E*loc had no significant differences across the three groups. Inter-group differences in basic network properties of structural networks including density, connectivity strength and size of largest component are shown in Supplementary Material (*SI Text*) and **Supplementary Figure S4**.

### Associations between Global Network Metrics and AVH Symptom Severity

For both functional and structural networks, we did not find any statistical correlations between global network metrics and ARHS total score in the AVH group.

## Discussion

To our knowledge, the present study is the first to investigate global topological alterations of functional and structural networks related to AVH in schizophrenia. We found that (1) schizophrenia patients with and without AVH exhibited common disruptions of functional networks, characterized by decreased clustering coefficient, global efficiency and local efficiency, and increased characteristic path length; (2) structural networks of only schizophrenia patients with AVH showed increased characteristic path length compared with those of healthy controls. These findings suggest that less “small-worldization” and lower network efficiency of functional networks may be an independent trait characteristic of schizophrenia, whereas regularization of structural networks may be the underlying pathological process engaged in schizophrenic AVH symptom expression.

The human brain is structurally and functionally organized into a complex network, the main feature of which is a small-world topology facilitating the segregation and integration of information processing (Bassett and Bullmore, 2006). Complex network analysis based on graph theory has been applied to quantify brain networks with a rich array of neurobiologically meaningful and easily computable metrics (Rubinov and Sporns, 2010), such as the most frequently used small-world properties and network efficiency. According to graph theory, the brain can be depicted as a graph composed of a number of nodes interconnected by a set of edges. The nodes represent brain regions or even voxels. The edges represent functional connectivity measured by resting-state fMRI or fiber bundles tracked by diffusion tensor tractography. Several functional (Salvador et al., 2005; Achard et al., 2006) and diffusion (Hagmann et al., 2007; Gong et al., 2009) MRI studies have consistently demonstrated that brain networks in healthy subjects have small-world topologies and high efficiency at a low wiring cost. A combined analysis of fMRI-based functional and DTI-based structural networks would provide integrated information on the underlying neuropathological mechanisms of brain disorders.

In the current study, we observed that functional networks of both schizophrenia patients with and without AVH had less “small-worldization” (characterized by lower local specialization and global integration) and lower network efficiency compared with the healthy controls, indicating that aberrant functional networks may be an trait characteristic of schizophrenia regardless of the presence or absence of AVH. Our findings are in line with several previous functional network studies. For example, Micheloyannis et al. (2006) found that patients with schizophrenia showed smaller absolute clustering coefficients but relatively longer absolute path lengths in the alpha, beta and gamma bands during resting-state using electroencephalogram (EEG). In a resting-state fMRI study, Liu et al. (2008) reported that functional networks of schizophrenic patients exhibited altered small-world attributes characterized by decreased clustering coefficient, increased characteristic path length and decreased small-worldness, as well as decreased global and local efficiency (Liu et al., 2008). Brain networks with small-world properties confer resilience against pathological attack and impairments of these properties may lead to the development of schizophrenia. These findings are also consistent with the increasing evidence that schizophrenia is a disorder of abnormal brain connectivity, that is, dysfunctional integration among multiple, spatially distributed brain regions (Fitzsimmons et al., 2013; van den Heuvel and Fornito, 2014; Fornito and Bullmore, 2015).

Compared with healthy controls, structural networks of schizophrenia patients showed no significant changes in the global network metrics, except that schizophrenia patients with AVH exhibited a lower global integration suggestive of a shift toward regular networks. A previous DTI study demonstrated that although a trend toward altered clustering, path length and small-worldness was found in schizophrenia patients, these differences were not significant (Zalesky et al., 2011), which is nearly consistent with our results. Different clinical profiles in patients (mixed vs. homogeneous) may account for the subtle differences between that study and ours. Our findings suggest that the regularization of structural networks might be the underlying neuropathology specific to AVH in schizophrenia. However, we did not find any correlations between global network metrics and AVH symptom severity, indicating that the network regularization process may be a stable pattern of schizophrenic AVH independent of its severity. However, we cannot exclude the possibility that there is a complex relationship between global network metrics and AVH symptom severity beyond a simple linear correlation.

The relationship between brain function and structure is traditionally illustrated by the hypothesis that neural activity is shaped, but not limited, by the underlying anatomy. In this study, however, converging evidence points out that functional and structural networks exhibit completely segregated changes related to AVH in schizophrenia. Functional networks are shown to be comprehensively disrupted and these impairments are specific to schizophrenia, indicating that functional network metrics are more sensitive biomarkers which can be used to detect whether schizophrenia exists. Structural networks present a slight regularization and this alteration is specific to AVH, indicating that structural network metrics are more specific biomarkers which can be used to predict and monitor AVH in schizophrenia.

There are several limitations to be considered in this study. First, most of the schizophrenia patients were receiving antipsychotic drug treatment. Although there was no difference in antipsychotic dosage between the schizophrenia patients with and without AVH, we cannot absolutely rule out the effect of antipsychotic medication on our results. Second, we did not assess the psychotic symptoms of patients during the MRI scan, which means that whether or not patients were experiencing AVH was unknown. Instead, AVH and other psychotic symptoms of all the schizophrenia patients were assessed before the MRI procedure. Third, the schizophrenic patients who had not experienced AVH within 12 months prior to MRI scanning were included in this study. These patients may have experienced AVH earlier in the psychotic illness, and they may still have trait characteristics of AVH. Fourth, the angular resolution of DTI was relatively low (25 directions) in this study, which may affect the robustness of the results of the tensor-based deterministic tracking method. Finally, we used AAL template to segment the whole brain into 90 regions for brain network construction. Recent studies have pointed out that the node definition by different parcellation strategies may lead to considerable variations in the graph theoretical metrics (Wang et al., 2009; Fornito et al., 2010; Hayasaka and Laurienti, 2010; Zalesky et al., 2010). In future studies, different parcellation schemes should be used to test the reproducibility of our findings.

## Conclusion

The present study used a combined analysis of functional and structural networks to investigate global topological alterations specific to AVH in schizophrenia. We found that functional networks of schizophrenia patients had less “small-worldization” and lower network efficiency regardless of the presence or absence of AVH. More importantly, only schizophrenia patients with AVH exhibited regularization of structural networks, which may be the underlying pathological mechanisms of AVH in schizophrenia. These findings suggest that functional network measures might be used to determine whether schizophrenia exists, and structural network measures can be used to predict and monitor AVH in schizophrenia.

## Author Contributions

JZ, CW, and CZ designed the study. JZ and JL acquired the data, which JZ, FL, and WQ analyzed. JZ, CW, and CZ wrote the article, which all authors reviewed and approved for publication.

## Conflict of Interest Statement

The authors declare that the research was conducted in the absence of any commercial or financial relationships that could be construed as a potential conflict of interest.

The reviewer MX and handling Editor declared their shared affiliation, and the handling Editor states that the process nevertheless met the standards of a fair and objective review.

## Footnotes

1 http://www.fil.ion.ucl.ac.uk/spm

2 http://www.nitrc.org/projects/gretna

3 http://www.fmrib.ox.ac.uk/fsl

4 http://trackvis.org/dtk
