# Supplementary material for: Alterations of Functional and Structural Networks in Schizophrenia Patients with Auditory Verbal Hallucinations
Source: Front Hum Neurosci. 2016 Mar 15;10:114. doi: 10.3389/fnhum.2016.00114 (PMC4791368; doi:10.3389/fnhum.2016.00114)
Supplement: Supplementary file 1 [file Presentation_1.PDF]

Supplementary Materials

SI Text

SI Results

Intergroup Differences in Basic Network Properties of Functional Networks

Compared with the control group, functional networks of nAVH group showed significantly decreased density and connectivity strength (Fig. S3). However, there were no significant differences in density and connectivity strength between AVH and control groups. In addition, we found that size of largest component had no significant difference across the three groups.

Intergroup Differences in Basic Network Properties of Structural Networks

As shown in Fig. S4, structural networks of both AVH and nAVH groups showed significantly decreased size of largest component relative to control group. Compared with the control group, structural networks of nAVH group exhibited significantly decreased density. However, there was no significant difference in density between AVH and control groups. Additionally, we found that connectivity strength had no significant difference across the three groups.

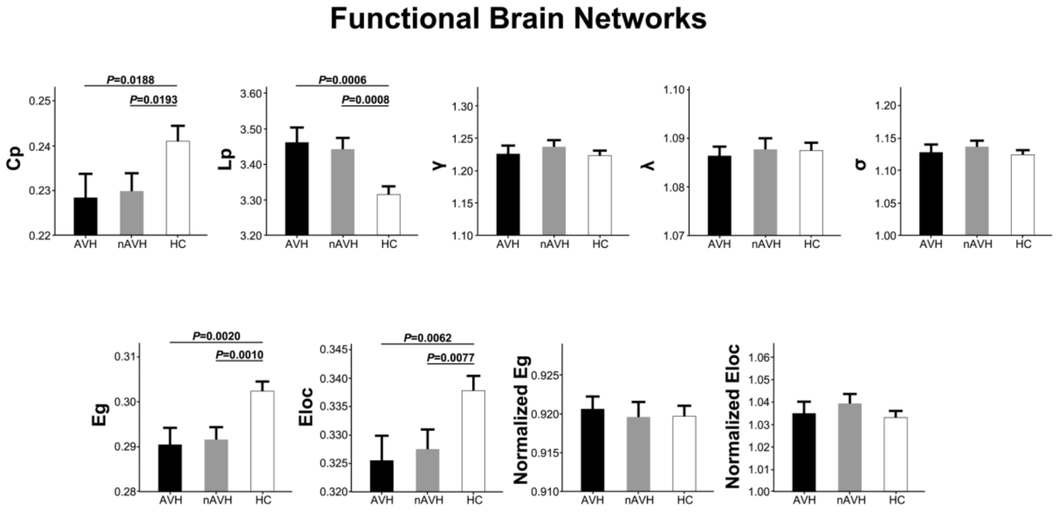

Figure S1. The inter-group differences in global metrics of the functional weighted networks constructed at a looser correlation threshold whose corresponding significance level survived a statistical threshold of $P < 0.001$ (uncorrected). Error bars represent standard errors. *Cp*, clustering coefficient; *Lp*, characteristic path length; γ , normalized clustering coefficient; λ , normalized characteristic path length; σ , small-worldness; *Eg*, global efficiency; *Eloc*, local efficiency; AVH, schizophrenia patients with auditory verbal hallucinations; nAVH, schizophrenia patients without auditory verbal hallucinations; HC, healthy controls.

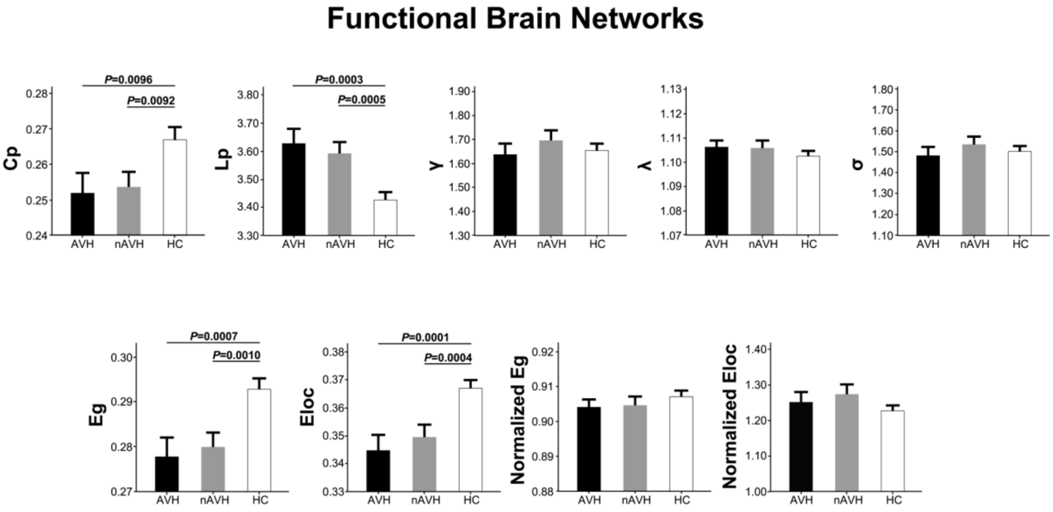

Figure S2. The inter-group differences in global metrics of the functional weighted networks constructed at a stricter correlation threshold whose corresponding significance level survived a statistical threshold of $P < 0.01$ (Bonferroni corrected). Error bars represent standard errors. *Cp*, clustering coefficient; *Lp*, characteristic path length; γ , normalized clustering coefficient; λ , normalized characteristic path length; σ , small-worldness; *Eg*, global efficiency; *Eloc*, local efficiency; AVH, schizophrenia patients with auditory verbal hallucinations; nAVH, schizophrenia patients without auditory verbal hallucinations; HC, healthy controls.

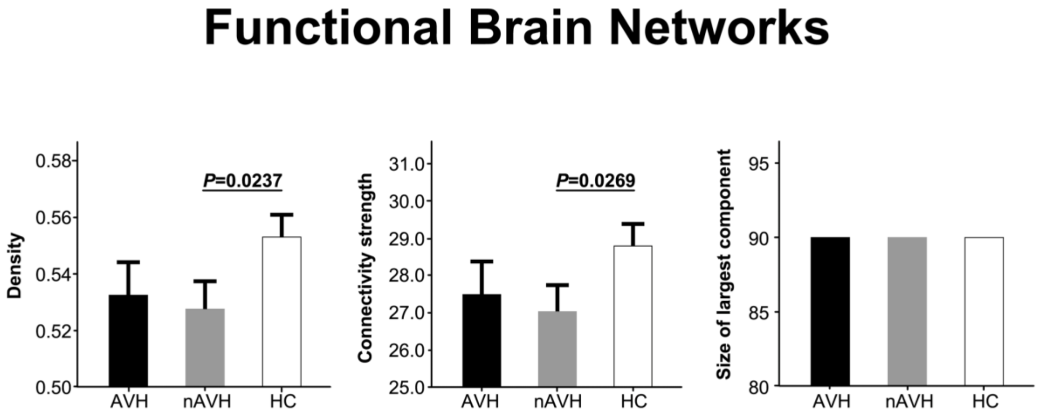

Figure S3. The inter-group differences in basic network properties of the functional weighted networks. Error bars represent standard errors. AVH, schizophrenia patients with auditory verbal hallucinations; nAVH, schizophrenia patients without auditory verbal hallucinations; HC, healthy controls.

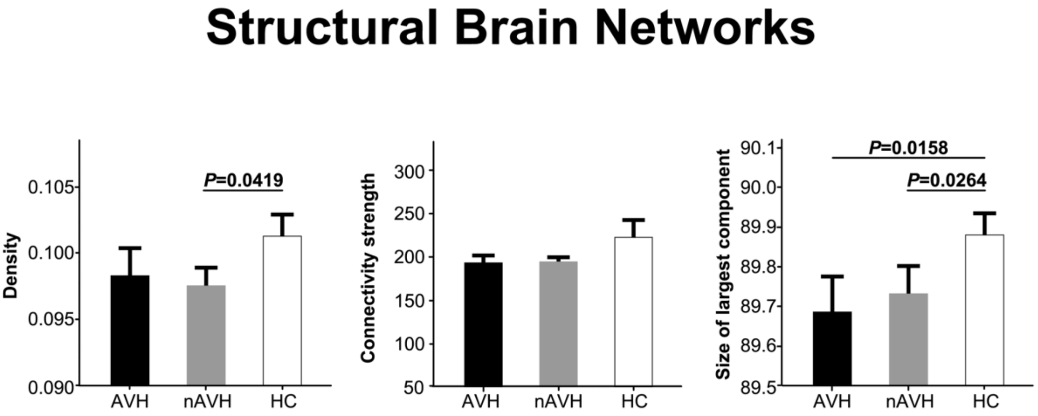

Figure S4. The inter-group differences in basic network properties of the structural weighted networks. Error bars represent standard errors. AVH, schizophrenia patients with auditory verbal hallucinations; nAVH, schizophrenia patients without auditory verbal hallucinations; HC, healthy controls.
